# Radial Tear of the Midbody in Complete Discoid Lateral Meniscus: A Case Report

**DOI:** 10.7759/cureus.75750

**Published:** 2024-12-15

**Authors:** Ryo Iuchi, Yoshiki Shiozaki, Shuji Horibe

**Affiliations:** 1 Department of Orthopedic Sports Medicine, Seifu Hospital, Sakai, JPN; 2 Department of Orthopedic Sports Medicine, Kansai Rosai Hospital, Amagasaki, JPN

**Keywords:** discoid lateral meniscus, meniscal repair, radial tear, surgical case report, tendon transplantation

## Abstract

To the best of our knowledge, there are no reports on the results of the repair of radial tears of the midbody of the complete discoid lateral meniscus (DLM). A 14-year-old female underwent meniscal replacement with autologous tendon transplantation for early re-tear after repair of the radial tear in the midbody of complete DLM. Two years after the tendon transplantation, there was no effusion or swelling, and the patient was able to exercise completely without symptoms. Repair without saucerization did not allow the placement of multiple sutures at the periphery and allowed destructive forces from the lateral femoral condyle to impact the tear site. Therefore, meniscectomy of the medial zone might be necessary to repair the radial tear of the midbody of complete DLM. Further follow-up is needed to conclude whether the tendon graft can replace the meniscus in the long term.

## Introduction

Discoid lateral meniscus (DLM), which is common in East Asia, has a unique structure [1], unlike the semilunar type; this results in a variety of DLM-specific injuries [2,3]. It has been previously reported that the subtotal or total meniscectomy under arthrotomy was performed without mentioning the injury type, and the long-term results were relatively good [4,5]. For the past 30 years, surgical treatment for torn DLMs has shifted to meniscoplasty (saucerization) [6-8] and the repair of peripheral tears with or without partial meniscectomy under arthroscopy to preserve the meniscal function as much as possible [9-11]. In most articles, the meniscal repair was performed for peripheral longitudinal tears [9-11]. On the other hand, there have been only a few reports on the repair of radial tears in the midbody of incomplete DLM [12,13], and to the best of our knowledge, there are no reports on the outcomes of meniscal repair of radial tears in the midbody of complete DLM. Hence, in this report, we present a case of a 14-year-old female with the repair of the radial tear in the midbody of complete DLM who underwent meniscal replacement with autologous tendon transplantation because of the early re-rupture of the repaired site.

## Case presentation

**Figure 1:**
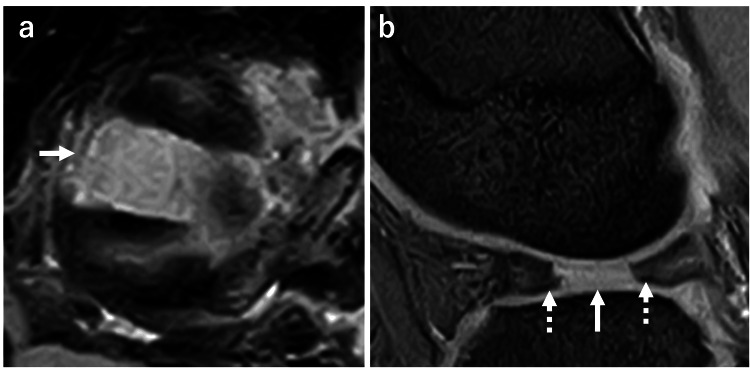
Preoperative MRI (a) Axial view showed a radial tear of the midbody of discoid lateral meniscus with an 11-mm-wide diastasis (white arrow) (b) Sagittal view showed a radial tear of the midbody and little degenerative change in the tear site (white dotted arrows)

A 14-year-old female basketball player injured her right knee during a game when she made a side-step cut with her knee flexed. Three days later, she visited our hospital with complaints of locking and pain in her right knee. The range of motion was restricted to 60°-120° due to severe pain in the lateral side of the knee. MRI revealed a radial tear in the midbody of complete DLM with an 11-mm-wide peripheral diastasis and slight degeneration in the meniscal body (Figure 1).

**Figure 2:**
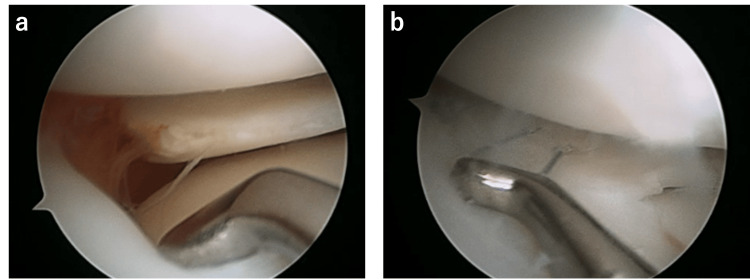
Meniscal repair (a) The complete radial tear of the midbody up to the peripheral rim (b) Repair using three stitches with trimming of the inner side

Arthroscopy was performed under general anesthesia 11 days post-injury, and it showed that the midportion of complete DLM was transversely torn with a large gap (Figure 2a). First, we tried performing meniscal repair without saucerization because we could easily reduce the tear site using a hook. After refreshing the torn site and adjacent synovium with a rasp to promote healing through an adequate vascular supply, the outer side of the tear was repaired with one stitch of #2-0 Fiberwire (Arthrex Japan, Tokyo, Japan). We used QuickPass SutureLasso 45°curve (Arthrex, Naples, FL) for the anterior stump and Scorpion (Arthrex, Naples) for the posterior stump. Moreover, the inner side was repaired with two stitches of #2-0 Fiberwire using a zone-specific cannula (Linvatec, Largo, FL). The repair site was sufficiently stable; therefore, the operation was performed without saucerization (Figure 2b), and the knee brace fixation was performed at 30°of knee flexion with no weight bearing.

**Figure 3:**
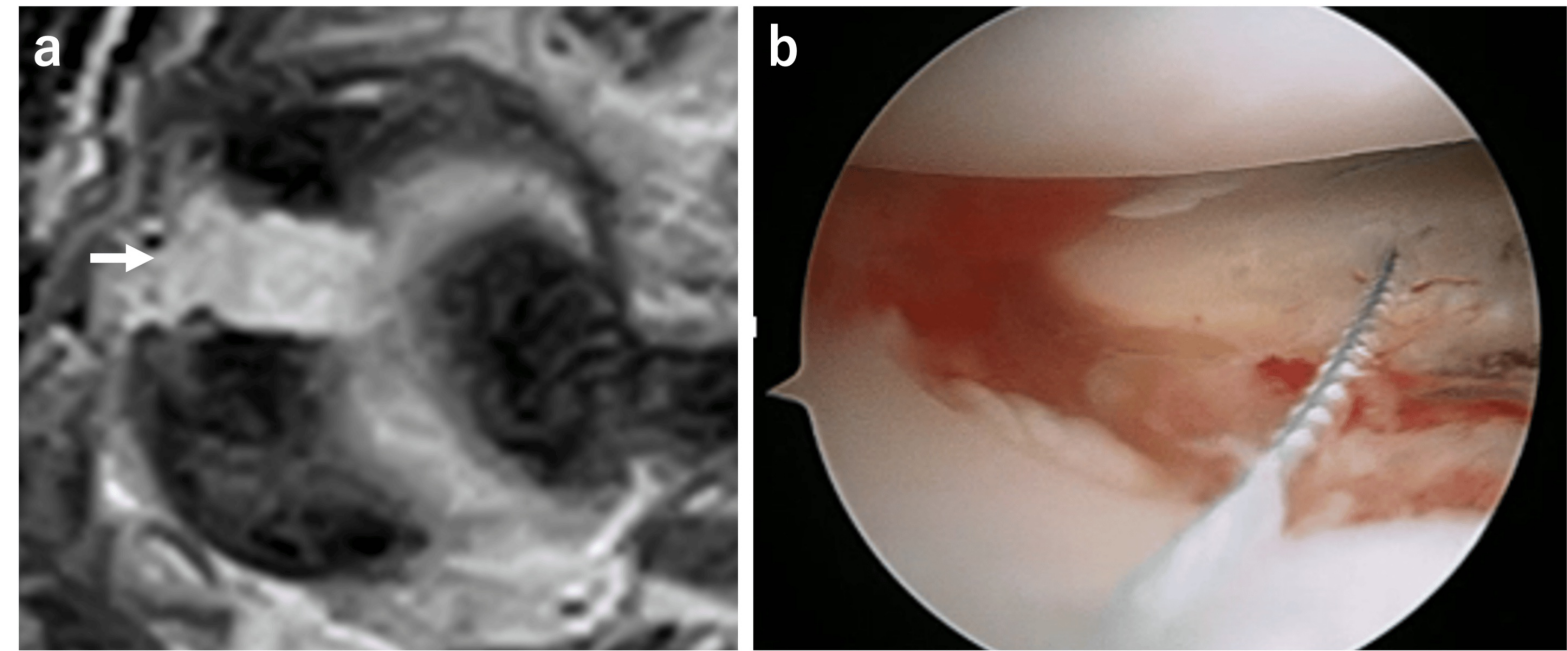
Failure of the repaired meniscus (a) Axial view on MRI showed re-diastasis of the repaired site (white arrow) (b) Rearthroscopy showed the broken repair site with the cutout of suture from the meniscus

**Figure 4:**
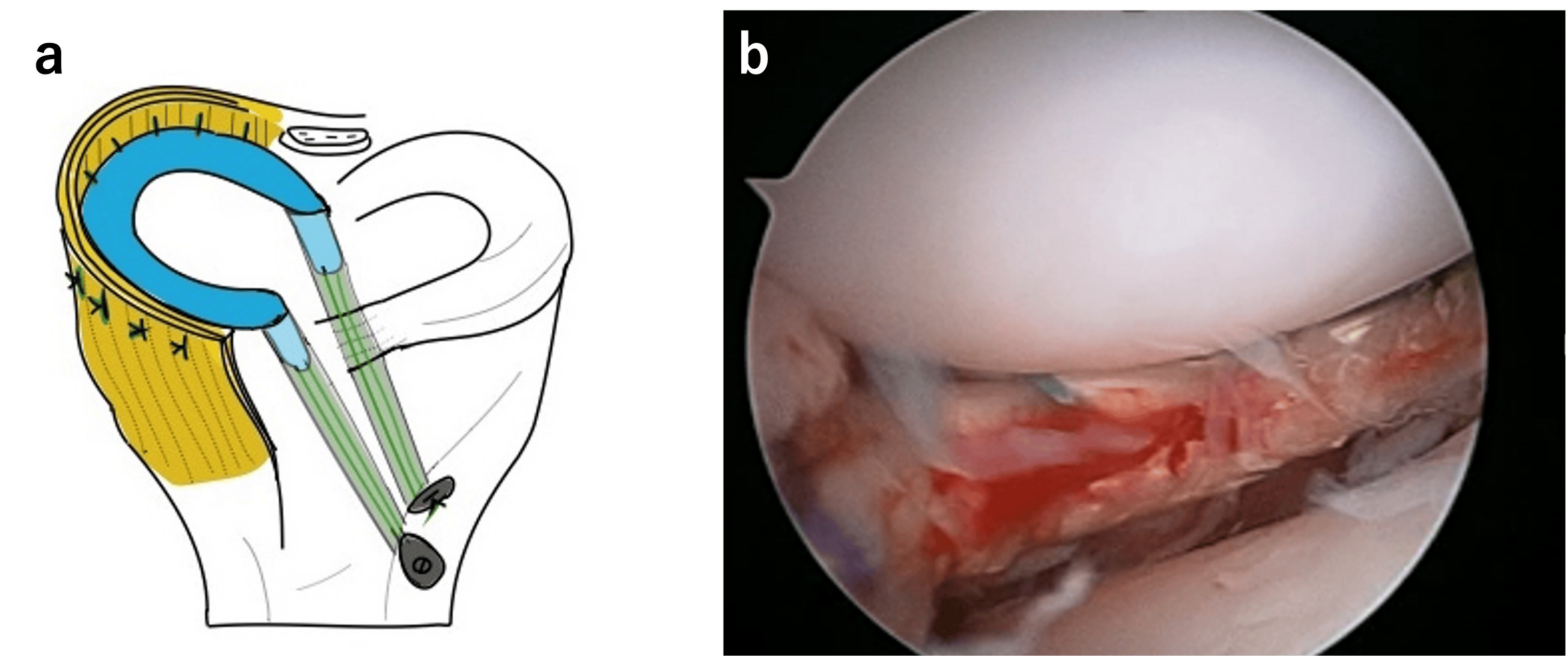
Meniscal replacement with autogenous semitendinosus tendon (a) Schema of tendon transplantation with autogenous semitendinosus tendon. (b) Semitendinosus tendon graft at the midportion of lateral joint space Panel A figure credits: Iuchi R

MRI was performed two days after surgery to check the repair site, and it showed the recurrence of separation (Figure 3a). We performed re-arthroscopy and found that the repaired site was broken with a cutout of the suture from the meniscus (Figure 3b). After subtotal meniscectomy, the semitendinosus tendon harvested from the ipsilateral lower limb was cut into 13 cm length, and care was taken to prevent the damage of epitenon. Two #2 Ultrabraid (Smith & Nephew, Andover, MA, USA) were placed at each end of the graft with Krackow suture, and a #2 PDS guide suture was placed at the center of the graft. By using an anterior cruciate ligament tibial drill guide (Smith & Nephew Endoscopy, Andover, MA), one 2.4 mm K-wire was inserted from the anteromedial aspect of the tibia to the anterior one-third of the anterior horn, and another 2.4 mm K-wire was inserted from the anteromedial aspect of the tibia to the posterior one-third of the posterior horn. Then, the guide wires were overdrilled using a 6 mm cannulated drill. A skin incision was made from the femoral lateral epicondyle to the Gerdy tubercle. A popliteal retractor was placed between the lateral head of the gastrocnemius and the posterior capsule to protect the peroneal nerve. The graft was introduced into the joint through a 5 mm incision at the joint line just anterior to the lateral collateral ligament, and it was fixed by tying the suture from the graft to the Endobutton (Smith & Nephew Endoscopy) after passing through the posterior hole. While maintaining tension on the graft by guide suture, the posterior half of the graft was sutured to the capsule with multiple vertical stacked sutures. After suturing the posterior half of the tendon to the capsule completely, the anterior half of the graft was inserted into the joint through the lateral aperture. Care was taken to avoid twisting of the graft, and the sutures were passed through the anterior tibial drill hole; #2-0 braided polyester sutures with zone-specific cannula were placed around the anterior half of the graft. Then, the sutures were tied to a Double Spike Plate (DSP) (MEIRA, Nagoya, Japan) (Figure 4) [14]. After the surgery, the knee was immobilized in a brace at 30° of flexion for three weeks. After the brace removal, the patient began active range of motion exercises. Partial weight-bearing was allowed at four weeks, and full weight-bearing was allowed after six weeks. The previous sports activities were allowed after six months.

**Figure 5:**
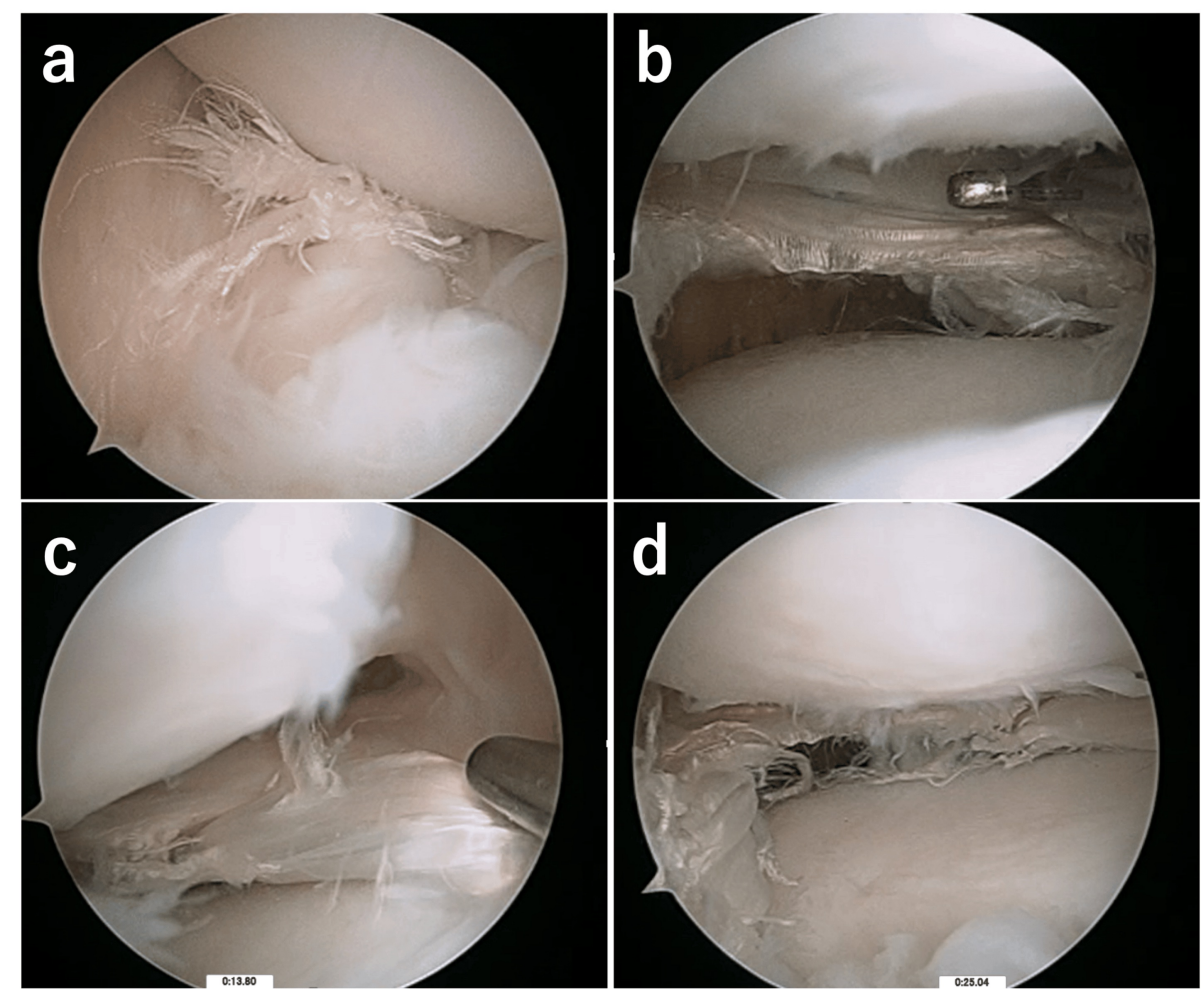
Second-look arthroscopy six months after tendon transplantation (a) Anterior portion; (b) midportion; (c) posterior portion; (d) whole image

**Figure 6:**
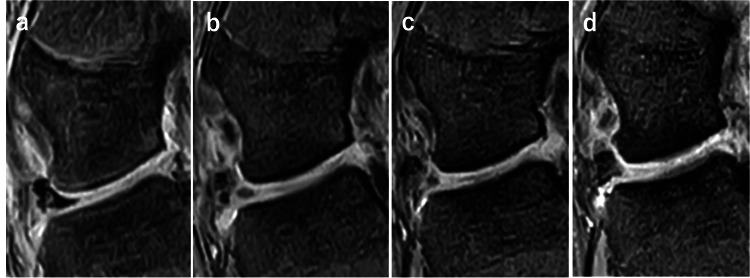
The change over time of the tendon graft on the coronal view of MRI (a) Post-operative at 10 days; (b) post-operative at three months; (c) post-operative at six months; (d) post-operative at two years.

**Figure 7:**
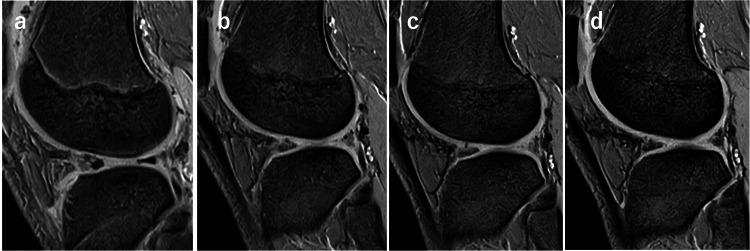
The change over time of the tendon graft on the sagittal view of MRI (a) Post-operative at 10 days; (b) post-operative at three months; (c) post-operative at six months; (d) post-operative at two years.

**Figure 8:**
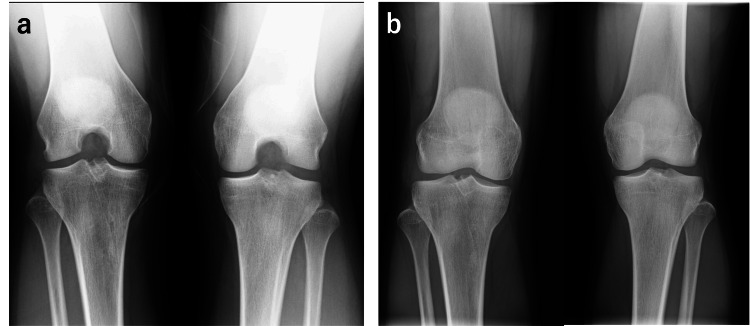
Radiographic findings two years after tendon transplantation (a) Rosenberg view, (b) standing anteroposterior view

MRI was performed 10 days postoperatively, and it showed that the meniscal graft had a similar morphology to the normal meniscus, whereas the width of the graft was smaller than that of the normal meniscus. T2-weighted MRI at three months postoperatively showed that the intensity of the graft was higher than that at 10 days postoperatively. Additionally, the coronal view showed that the meniscal graft was laterally extruded in the middle segment. The intensity of the graft became higher on T2-weighted MRI performed six months postoperatively (Figures 5-6). Rearthroscopy showed that the volume of the graft in the middle segment was smaller than that in the anterior and posterior segments, whereas the healing between the graft and capsule was obtained in all segments (Figure 5). Six months postoperatively, the patient returned to basketball without pain, swelling, and restriction in range of motion. Two years postoperatively, the intensity of the meniscal graft became lower without the progression of the extrusion in the middle segment and the change of graft volume on MRI (Figures 6-7). The Rosenberg view showed a narrowing of the joint space with a small spur on the lateral side of the tibia (Figure 8).

## Discussion

To the best of our knowledge, there are no reports on the repair of the radial tear of the midbody of complete DLM, and subtotal resection is usually performed for such tears. According to previous reports, the outcomes after the subtotal resection of DLM are not poor compared to those of the semilunar type meniscus [4,5]. Habata et al. reported satisfactory clinical results and mild arthritic changes in young patients with an average of 14.5-year follow-up for total resection of DLM [5]. However, ideally, the torn meniscus should be preserved as much as possible.

Recently, meniscal repair has been actively attempted for radial tears in the midbody of the lateral meniscus [12,13,15]. Thus, we also tried to repair the radial tear of complete DLM using hybrid all-inside repair techniques and conventional inside-out suture techniques. As the repair site was sufficiently stable, we decided not to perform saucerization in order to preserve as much meniscal tissue as possible and to trim the poorly healed area by re-arthroscopy at six months postoperatively. However, the repaired meniscus resulted in failure immediately after the surgery. This failure might be due to the structural difference between complete DLM and semilunar type meniscus. The peripheral collagen fibrils are displayed as dense bundles running in a circumferential pattern, whereas the medial zone shows thin, loosely, and irregularly arranged fibrils without bundle formation [1]. In this report, only one stitch was threaded into the peripheral fibrils, whereas two stitches were threaded into the fragile medial zone. Therefore, the repair in this report could not sufficiently secure the stabilization of the torn site.

Additionally, the contact of the lateral femoral condyle (LFC) to the meniscus might give a destructive force to the torn site and lead to the separation of the torn site. Preoperative MRI scanned in a neutral position of the varus-valgus of the knee showed the distal part of LFC located in the torn site (Figure 1), whereas this contact could not be detected during the meniscal repair because of the widening of joint space by varus position of the knee (Figure 2). Therefore, the meniscectomy of the medial zone might be necessary to reduce the LFC contact. Following this, repair with multiple stitches to the peripheral rim might be more conducive to achieving stronger repair. Hence, repair without saucerization has been reported in recent years for peripheral longitudinal tears of the complete DLM [11], whereas it may not be indicated for the complete radial tear in the midbody of the complete DLM.

Treatment options after the failure of the repaired meniscus are as follows: (1) observation without surgery, (2) subtotal meniscectomy alone, (3) immediate meniscal replacement after subtotal meniscectomy, or (4) re-repair with saucerization. After an extensive discussion about the treatment plan with the patient and her parents, we chose subtotal meniscectomy followed by immediate meniscal replacement with autologous tendon transplantation.

Meniscal allograft transplantation has been generally used for replacement. Kim et al. performed 14 meniscal allografts that were implanted with a previous total or near-total resection of torn DLM; they reported that symptoms improved in all cases with an increment of the modified Lysholm score (71.4-91.4) for a minimum of 21 months of follow-up and normal appearance on second-look arthroscopy in four of six knees [16]. However, in Japan, the meniscal allograft is unavailable due to the medical system and religious matters [14]. As an alternative method of meniscal transplantation, Kohn et al. (1992) reported successful transplantation of the autogenous patellar tendon in a sheep model [17]. Encouraged by this animal experiment, several clinical studies have been reported [14,18,19]; however, the results have been unfavorable. In these studies, tendon transplantation was performed for cases with preoperative osteoarthritis, anterior instability, or malaligned leg, which were not ideal for remodeling of tendon graft and meniscal allograft transplantation. Although it is an animal study, Aagard et al. reported that immediate meniscal allograft transplantation was superior to delayed transplantation after meniscectomy and meniscectomy alone in preventing cartilage degeneration [20]. Therefore, we hypothesized that this case, which resulted in failure immediately after meniscal repair, was best suited for tendon transplantation, and that transplantation would lead to a better result than subtotal meniscectomy alone to prevent cartilage degeneration. Therefore, we performed tendon transplantation immediately after resection.

Consequently, there were no symptoms, and little degenerative change was seen on X-ray two years after surgery. However, further follow-up is necessary to conclude whether the tendon graft would play a role as an alternative to meniscus in the long term.

## Conclusions

We present the case of a 14-year-old female who underwent meniscal replacement with autologous semitendinosus tendon transplantation for early re-tear after repair of the radial tear in the midbody of complete discoid lateral meniscus. The failure to successfully repair the meniscus tear without saucerization may be attributed to the structural and morphological differences between the complete DLM and the semilunar meniscus. Meniscectomy of the medial zone might be necessary for the repair of the radial tear of the midbody of complete DLM. Although the patient had no symptoms two years after the tendon graft, further follow-up is needed to conclude whether a tendon graft can replace the meniscus in the long term.
